# *FCGR2A, FCGR3A* polymorphisms and therapeutic efficacy of anti-EGFR monoclonal antibody in metastatic colorectal cancer

**DOI:** 10.18632/oncotarget.4872

**Published:** 2015-08-19

**Authors:** Hou-Qun Ying, Feng Wang, Xiao-Lin Chen, Bang-Shun He, Yu-Qin Pan, Jie Chen, Xian Liu, Wei-Jun Cao, Hong-Xin Peng, Kang Lin, Shu-Kui Wang

**Affiliations:** ^1^ Medical College, Southeast University, Nanjing 210009, Jiangsu, China; ^2^ Central Laboratory, Nanjing First Hospital, Nanjing Medical University, Nanjing 210006, Jiangsu, China; ^3^ Life Scientific College, Nanjing Normal University, Nanjing 210046, Jiangsu, China; ^4^ Department of Clinical Laboratory, Pingxiang People's Hospital, Pingxiang, 337055, Jiangxi, China; ^5^ Department of Digestion, Nanjing First Hospital, Nanjing Medical University, Nanjing 210006, Jiangsu China

**Keywords:** H131R, F158V, FCGR, mCRC, anti-EGFR mAb

## Abstract

Anti-EGFR monoclonal antibodies (mAb) such as cetuximab, panitumumab are one kind of efficacious targeted drugs in treatment of metastatic colorectal cancer (mCRC). However, only a small proportion of patients harbored wild-*KRAS* genotype can benefit from it. We hypothesized that personal genetic heterogeneity might be the main cause leading to obvious difference in its clinical efficacy. A retrospective study including 82 mCRC patients treated with chemotherapy plus cetuximab and a comprehensive meta-analysis containing 2831 cases within sixteen eligible studies were conducted to investigate the possible association between *FCGR2A* H131R and *FCGR3A* V158F and clinical outcome of mCRC patients treated with anti-EGFR mAb based therapy. Results of the retrospective study showed that H131R within *FCGR2A* or V158F within *FCGR3A* were not associated with clinical outcome in 82 *KRAS* wild chemorefractory mCRC patients in co-dominant, dominant, recessive, over-dominant, allele genetic models. However, the comprehensive meta-analysis with the largest of sample size obtained the significant result between *FCGR3A* V158F and PFS (FV/VV vs. FF: *P*_h_ = 0.027, MSR = 0.680, 95%CI = 0.549−0.842 in overall population; *P*_h_ = 0.12, MSR = 0.728, 95%CI = 0.648–0.818 in *KRAS* wild population) and OS (VV vs. FF: *P*_h_ < 0.001, MSR = 0.733, 95%CI = 0.578−0.930 in overall population). These findings indicate that *KRAS* wild chemorefractory mCRC individual harbored genotype FF of V158Fcan benefit from anti-EGFR mAb adjuvant therapy in terms of PFS and OS, and it may be useful genetic biomarker to predict clinical survival of mCRC individuals with anti-EGFR mAb based therapy.

## INTRODUCTION

Colorectal cancer (CRC) is one of the most common lethal malignancies in the United States and China [1, 2]. With limitation of useful early diagnostic biomarker and invasiveness of colonoscopy, most of cases are clinical confirmed as TNM II-IV patients, and mid-term patients can benefit from regular surgical operation plus adjuvant chemoradiotherapy. Meanwhile, the therapeutic options against metastatic CRC (mCRC) have expanded with the introduction of anti-epidermal growth factor receptor monoclonal antibody (anti-EGFR mAb) and treatment outcomes have improved in the past decade [3].

Cetuximab, a chimeric immunoglobulin 1 monoclonal antibody that targets the extracellular domain of the EGFR, has been validated to be effective in providing clinical benefit in wild-*KRAS* mCRC patients in combination with chemotherapy as first and second lines as well as in monotherapy as third line drug [3–7]. However, only approximately 10~20% of patients with chemorefractory mCRC derived good clinical benefit from cetuximab therapy [8], revealing that personalized difference in genetic background might affect individual's response and additional mechanisms could lead to CRC progression [9]. Therefore, an understanding of molecular basis of clinical response to cetuximab may be better to identify the subpopulation of patients who are likely to benefit from cetuximab and avoid unnecessary drug toxicity and costs.

The molecular mechanisms underlying response to cetuximab are still substantially unclear; it is that cetuximab acts by means of inhibition of EGFR signal pathway or activation of antibody-dependent cell cytotoxicity (ADCC) [10]. However, only 30~40% non-responsive patients harbored *KRAS* mutation [11, 12], and some *KRAS* mutated patients also showed to respond to cetuximab [13]. These exacting evidences suggested that ADCC might be involved in cetuximab enhanced antitumor efficacy [13]. ADCC is stimulated through the interaction between Fc fragment of lgG1 monoclonal antibodies linked with EGFR of targeted cancer cell and the surface Fc gamma receptor 2a and 3a (FCGR2A and FCGR3A) of IgG carried by immune cells such as natural killer lymphocytes (NK), macrophages and neutrophil, triggering the activation of these immune effective cells and leading to the lysis and death of targeted cancer cell. Genetic variations within *FCGR2A* and *FCGR3A* may contribute to abnormal secondary spatial structure and function of the products, leading to different binding affinity to cetuximab. H131R and V158F are two common single nucleotide polymorphisms (SNPs) which are located in the third and fifth exon of *FCGR2A* and *FCGR3A*, respectively, and two loci appear to be associated with clinical outcome in hematological malignancies and breast cancer with treatment of rituximab and trantuzumab, respectively [14, 15]. Follicular lymphoma patients harbored allele V and genotype VV of V158F within *FCGR3A* could benefit from the combination therapy including rituximab [16] and genotype HH of H131R within *FCGR2A* was significantly associated with a shorter event-free survival in breast cancer patients with sequentially given transtuzumab in UNICANCER-PASCO4 trial [17]. Some studies attempted to investigate the role of the two SNPs in treatment efficacy of anti-EGFR mAb in advanced CRC patients, however, these results weren't in consistence with each other [18–20].

In this study, we aimed to investigate the association of *FCGR2A* H131A and *FCGR3A* V158F with clinical outcome of 82 wild-*KRAS* chemorefractory mCRC patients undergoing cetuximab adjuvant therapy. Additionally, a comprehensive meta-analysis including prospective and retrospective studies was carried out to confirm the clinical finding.

## RESULT

Overall, a total of 46 male and 36 female chemorefractory mCRC individuals harbored wide-*KRAS* were included in our study. 52 and 30 were colon and rectal cancer patients, respectively. All of them were TNM-IV stage patients and treated with chemotherapy plus cetuximab. However, only 6 CR, 44 PR, 15 SD and 17 PD were observed in 82 mCRC individuals, respectively. The genotype distributions of H131R within *FCGR2A* and V158F within *FCGR3A* were in Hardy-Weinberg equilibrium (*P* = 0.52 for *FCGR2A*, and *P* = 0.09 for *FCGR3A*, respectively) (Table 1).

**Table 1:** Baseline characteristics of 82 mCRC patients treated with adjuvant cetuximab

Characteristic	No. of patients	%
Median and range of age (years)	61 (51–70)	
Gender		
Male/female	46/36	56.1%/43.9%
Location		
Left/right colon/rectal	25/27/30	30.5%/32.9%/36.6%
Treatment		
Cetuximab + CapeOX	8	9.76%
Cetuximab + CapeOX+AVASTIN	2	2.43%
Cetuximab +FOLFIR1	19	23.17%
Cetuximab +FOLFIR1+AVASTIN	1	1.22%
Cetuximab +FOLFIRI+ Capecitabine	1	1.22%
Cetuximab+FOLFOX	42	51.23%
Cetuximab+FOLFOX+FOLFIRI	5	6.10%
Cetuximab+FOLFOX+AVASTIN	1	1.22%
Cetuximab+Tegafur	1	1.22%
Cetuximab+Capecitabine	2	2.43%
Tumor response		
CR/PR	6/44	7.32%/53.66%
SD/PD	15/17	18.29%/20.73%
*FCGR2A* H131R		
HH/HR/RR	33/44/5	40.24%/53.66%/6.10%
*FCGR3A* F158V		
FF/FV/VV	49/25/8	59.76%/30.49%/9.75%

**Abbreviation:** mCRC: metastatic colorectal cancer; CR: complete response; PR: partial response; SD: stable disease; PD: progressive disease; CapeOX: capecitabine plus oxaliplatin; FOLFIR1: 5-fluorouracil plus calcium folinate plus irinotecan; FOLFOX: 5-fluorouracil plus calcium folinate plus oxaliplatin.

The influence of the two SNPs on the clinical efficacy and survival were described in Table 2 and 3. H131R within *FCGR2A* weren't associated with ORR (*P* = 0.542 for HR vs. HH; *P* = 0.357 for RR vs. HH; *P* = 0.454 for HR/RR vs. HH; *P* = 0.598 for RR vs. HH/HR; *P* = 0.710 for HR vs. HH/RR; *P* = 0.409 for R vs. H) and DCR (*P* = 0.644 for HR vs. HH; *P* = 0.461 for RR vs. HH; *P* = 0.559 for HR/RR vs. HH; *P* = 0.527 for RR vs. HH/HR; *P* = 0.787 for HR vs. HH/RR; *P* = 0.510 for R vs. H) in co-dominant, dominant, recessive, over-dominant and allele genetic models, respectively. No statistical significant difference in response to cetuximab based therapy (*P* = 0.425 for FV vs. FF; *P* = 0.835 for VV vs. FF; *P* = 0.454 for FV/VV vs. FF; *P* = 0.967 for VV vs. FF/FV; *P* = 0.441 for FV vs. FF/VV; *P* = 0.535 for V vs. F) or DCR (*P* = 0.463 for FV vs. FF; *P* = 0.957 for VV vs. FF; *P* = 0.559 for FV/VV vs. FF; *P* = 1.000 for VV vs. FF/FV; *P* = 0.446 for FV vs. FF/VV; *P* = 0.718 for V vs. F) based on *FCGR3A* V158F was observed. Also, there was no significant association between *FCGR* combined genotype and ORR (*P* = 0.642 for RR or VV vs. H and F) and DCR (*P* = 0.554 for RR or VV vs. H and F) in present study.

**Table 2:** Clinical response of wild-*KRAS* mCRC patients treated with adjuvant cetuximab according to *FCGR2A* and *FCGR3A* polymorphisms

Genetic Model	Polymorphism	No. of patients	Objective response			Disease control		
			CR+PR	SD+PD	*P*-value	CR+PR+SD	PD	*P*-value
	***FCGR2A* H131R**		No.	No.		No.	No.	
Co-dominant	HR vs. HH	44/33	12/7	32/26	0.542	21/14	23/19	0.644
	RR vs. HH	5/33	2/7	3/26	0.357*	3/14	2/−19	0.461*
Dominant	HR/RR vs. HH	49/33	14/7	35/26	0.454	24/14	25/19	0.559
Recessive	RR vs. HH/HR	5/77	2/19	3/58	0.598*	3/35	2/42	0.527*
Over-dominant	HR vs. HH/RR	44/38	12/9	32/29	0.71	21/17	23/21	0.787
Allele	R vs. H	54 /110	16/26	38/84	0.409	27/49	27/61	0.51
	***FCGR3A* F158V**							
Co-dominant	FV vs. FF	25/49	5/14	20/35	0.425	10/24	15/25	0.463
	VV vs. FF	8/49	2/14	6/35	0.835*	4/24	4/25	0.957*
Dominant	FV/VV vs. FF	33/49	7/14	26/35	0.454	14/24	19/25	0.559
Recessive	VV vs. FF/FV	8/74	2/19	6/55	0.967*	4/34	4/40	1.000*
Over-dominant	FV vs. FF/VV	25/57	5/16	20/41	0.441	10/28	15/29	0.446
Allele	V vs. F	41/123	9/33	32/90	0.535	18/58	23/65	0.718
	***FCGR* combined**							
	H and F vs RR or VV	69/13	17/4	52/9	0.642	31/7	38/6	0.554

**Abbreviation:** mCRC: metastatic colorectal cancer; CR: complete response; PR: partial response; SD: stable disease; PD: progressive disease. *P*-value: result of chi-square test;

* *P*-value: result of fisher's exact test.

**Table 3:** The polymorphisms of *FCGR2A* and *FCGR3A* and clinical survival of 82 wild-*KRAS* mCRC patients treated with adjuvant cetuximab

Model	Locus	Progression-free survival					Overall survival				
		Months		*P*-value*	Cox		Months		*P*-value*	Cox	
		Median	95%CI		HR^#^	95%CI	Median	95%CI		HR^#^	95%CI
***FCGR2A* H131R**											
Co-dominant	HR vs. HH	7/5.5	5.22−8.78/1.27−9.73	0.937	1.086	0.636−1.856	13/13	10.54−15.46/8.43−17.57	0.642	1.332	0.765−2.318
	RR vs. HH	6/5.5	3.52−8.48/1.27−9.73	0.675	0.608	0.203−1.816	13/13	11.76−14.24/8.43−17.57	0.247	1.341	0.474−3.797
Dominant	HR/RR vs. HH	6/5.5	5.19−6.81/1.27−9.73	0.996	1.02	0.608-1.713	13/13	11.80−14.20/8.43−17.57	0.49	1.329	0.779−2.269
Recessive	RR vs. HR/HH	6/6	3.52−8.48/5.16−6.84	0.586	0.636	0.223−1.815	13/13	11.76−14.24/11.66−14.34	0.249	1.233	0.475−3.203
Over-dominant	HR vs. HH+RR	7/6	5.22−8.78/4.83−7.17	0.824	1.162	0.687−1.964	13/13	10.54−15.46/12.24−13.77	0.866	1.239	0.726−2.113
Allele	R vs. H	6/6	4.72−7.28/4.38−7.62	0.86	0.989	0.733−1.333	13/13	11.18−14.82/10.58−15.42	0.357	1.191	0.870−1.631
***FCGR3A* V158F**											
Co-dominant	FV vs. FF	6/8	4.62−7.39/4.78−11.23	0.619	0.845	0.453−1.577	14.5/14	10.21−18.79/11.04−16.96	0.777	1.002	0.472−2.127
	VV vs. FF	5.5/8	0.00−11.04/4.78−11.23	0.933	0.936	0.406−2.159	12/14	2.50−21.50/11.04−16.96	0.815	0.828	0.344−1.996
Dominant	FV/VV vs. FF	6/8	4.38−7.62/4.78−11.23	0.69	0.801	0.470−1.365	14.5/14	9.39−19.61/11.04−16.96	0.724	0.901	0.495−1.642
Recessive	VV vs. FF/FV	5.5/7	0.00−11.04/4.54−9.46	0.852	0.839	0.375−1.877	12/14	2.50−21.50/10.98−17.02	0.698	0.797	0.349−1.819
Over-dominant	FV vs. FF/VV	6/8	4.62−7.39/5.26−10.74	0.768	0.861	0.469−1.581	14.5/14	10.21−18.79/11.78−16.22	0.914	1.06	0.515−2.184
Allele	V vs. F	6/8	4.18−7.83/5.68−10.32	0.682	0.862	0.581−1.278	14.5/14	8.68−20.32/11.87−16.13	0.632	0.888	0.585−1.349
***FCGR* combined**											
H and F vs RR or VV		6/9	3.92−8.08/ 6.87−11.13	0.398	0.798	0.364−1.750	13/15	10.21−15.79/ 12.51−17.49	0.903	0.823	0.360−1.878

**Abbreviation:** mCRC: metastatic colorectal cancer;

* *P*-value: Result of log-rank test of Kaplan-Meier curve;

# HR: hazard ratio adjusted by sex, age, smoking, drinking and status of diabetes and hypertension; CI: confidential interval.

**Figure 1:**
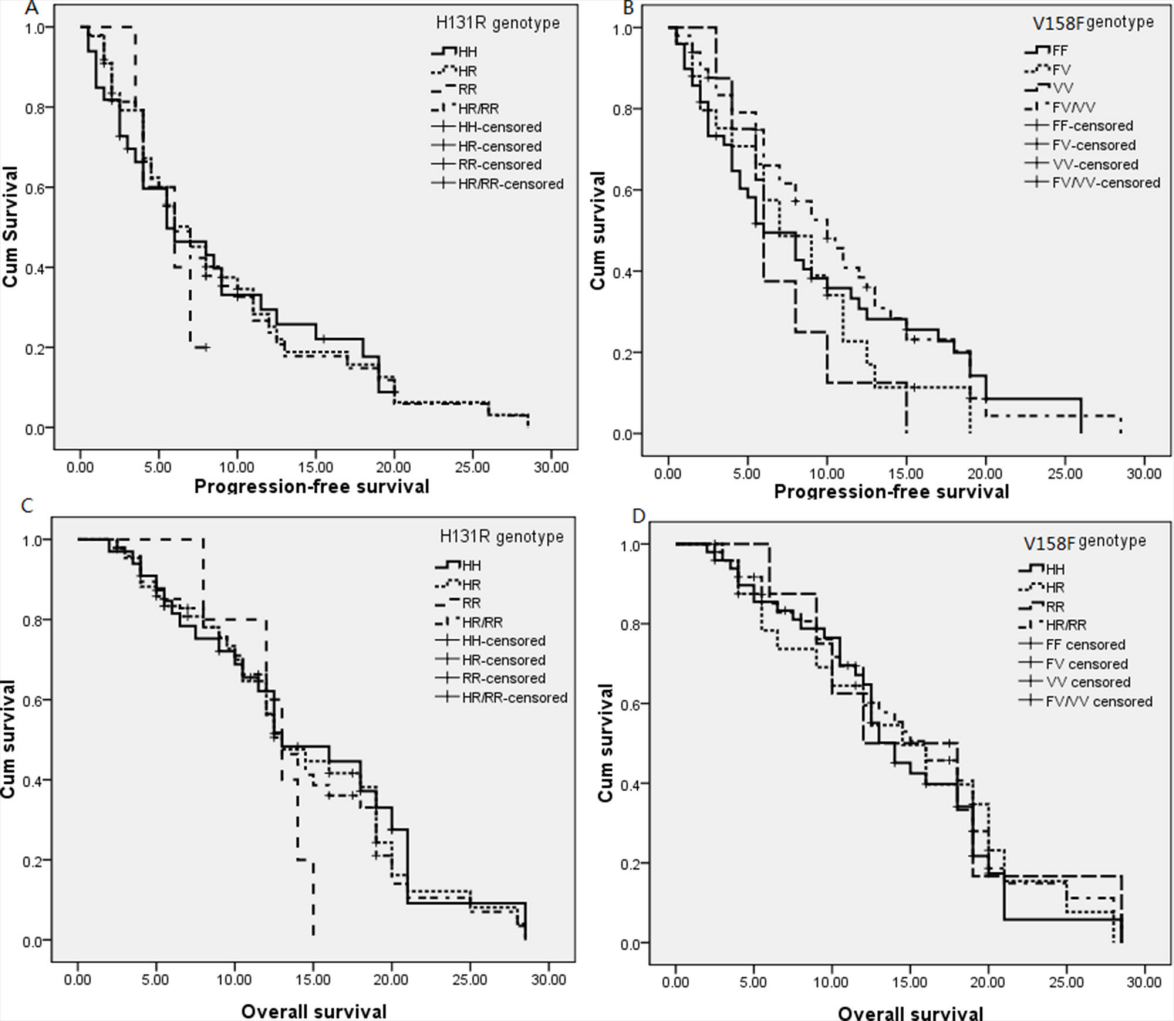
Kaplan-Meier curves for *FCGR2A* H131R and *FCGR3A* V158F for progression-free survival and overall survival **A.** H131R for PFS; **B.** V158F for PFS; **C.** H131R for OS; **D.** V158F for OS.

The median PFS of cases harbored allele R and H within H131R of *FCGR2A* were 6.0 and 6.0 months, and 5.5, 7.0, 6.0, 6.0, 6.0, 6.0 months for genotype HH, HR, RR, HR/RR, HH/HR, HH/RR of *FCGR2A*, respectively. However, H131R wasn't associated with PFS in co-dominant (HR = 1.086, 95%CI = 0.636–1.856 for HR vs. HH; HR = 0.608, 95%CI = 0.203–1.816 for RR vs. HH), dominant (HR = 1.02, 95%CI = 0.608–1.713), recessive (HR = 0.636, 95%CI = 0.223–1.815), over-dominant (HR = 1.162, 95%CI = 0.687–1.964) and allele (HR = 0.989, 95%CI = 0.733–1.333) models. The median OS of cases carrying H131R genotypes and alleles was 13 months, and there was no significant difference in OS in comparison of HR vs. HH (HR = 1.332, 95%CI = 0.765–2.318), RR vs. HH (HR = 1.341, 95%CI = 0.474–3.797), HR/RR vs. HH (HR = 1.329, 95%CI = 0.779–2.269), RR vs. HR/HH (HR = 1.233, 95%CI = 0.475–3.203), HR vs. HH/RR (HR = 1.239, 95%CI = 0.726–2.113), allele R vs. H (HR = 1.191, 95%CI = 0.870–1.631), respectively. Patient harbored genotype FV (HR = 0.845, 95%CI = 0.453–1.577 for PFS, HR = 1.002, 95%CI = 0.472–2.127 for OS), VV (HR = 0.936, 95%CI = 0.406–2.159 for PFS, HR = 0.828, 95%CI = 0.344–1.996 for OS) and FV/VV (HR = 0.801, 95%CI = 0.470–1.365 for PFS, HR = 0.901, 95%CI = 0.495–1.642 for OS) of V158F within *FCGR3A* were not shown a statistically longer or shorter PFS and OS than those individuals harbored genotype FF, respectively, no significant association was observed in recessive (HR = 0.839, 95%CI = 0.375–1.877 for PFS; HR = 0.797, 95%CI = 0.349–1.819 for OS), over-dominant (HR = 0.861, 95%CI = 0.469–1.581 for PFS; HR = 1.06, 95%CI = 0.515–2.184 for OS), allele (HR = 0.862, 95%CI = 0.581–1.278 for PFS; HR = 0.888, 95%CI = 0.585–1.349 for OS) models. Meanwhile, PFS (HR = 0.798, 95%CI = 0.364–1.750) and OS (HR = 0.823, 95%CI = 0.360–1.878) of the cases harbored genotype RR or VV weren't shown significant difference when compared to cases with allele H and F (Table 3, Figure 1).

**Figure 2:**
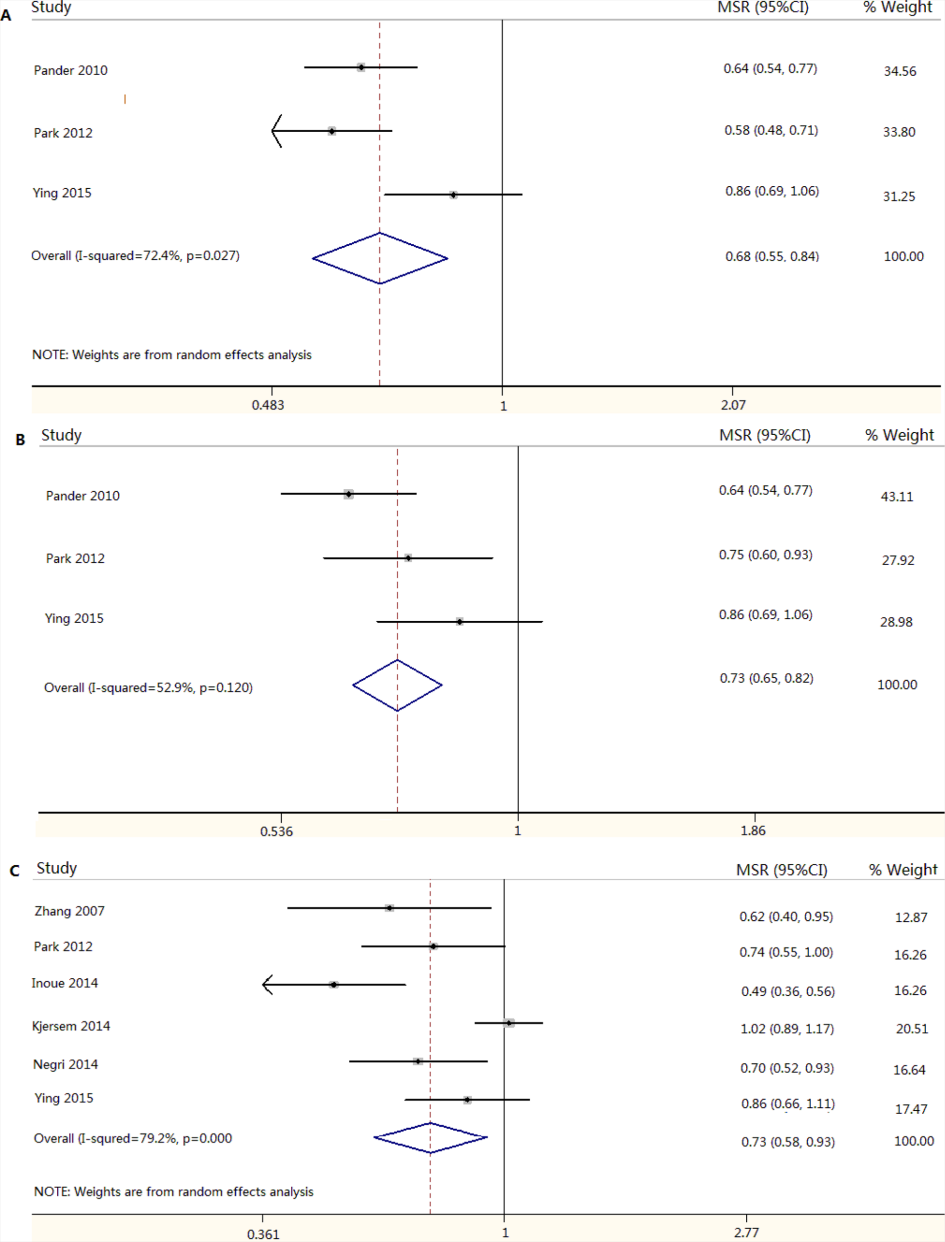
Meta-analysis result of association between *FCGR3A* V158F and progression-free survival and overall survival in mCRC patients treated with anti-EGFR mAb based therapy **A.** FV/VV vs. FF for PFS in overall population; **B.** FV/VV vs. FF for PFS in *KRAS* wild population; **C.** VV vs. FF for OS in overall population.

A total of 14 published articles (15 eligible studies) and our study were included in this comprehensive meta-analysis to further evaluate the association of *FCGR2A* and *FCGR3A* polymorphisms with clinical outcome in advanced CRC patients undergoing anti-EGFR mAb based therapy [18–31]. The baseline characteristics of included studies and the results of heterogeneity test, odds ratio (OR), median survival ratio (MSR) and corresponding 95% confidential interval (95%CI) were reported in Supplementary Table 1 and Table 4 and 5. As shown from Table 4, Genotypes of H131R weren't associated with clinical outcome of overall and *KRAS* wild chemorefractory mCRC patients in terms of ORR, DCR in co-dominant, dominant, recessive, over-dominant, allele models, PFS and OS in co-dominant and dominant models. No significant difference was observed between ORR or DCR and genotypes and alleles of V158F, whatever the *KRAS* status. However, genotype FV/VV within V158F of *FCGR3A* was observed to be significant associated with a shorter PFS in overall (MSR = 0.680, 95%CI = 0.549–0.842) and *KRAS* wild population patients (MSR = 0.728, 95%CI = 0.648–0.818), and individuals harbored genotype FF showed a longer OS than those carrying genotype VV of *FCGR3A* V158F only in overall population (MSR = 0.733, 95%CI = 0.578–0.930) (Table 5 and Figure 2). There was no significant publication bias in all comparisons between genotypes of H131R and V158F and clinical response and outcome, respectively.

**Table 4:** Meta-analysis results of the association between *FCGR2A* H131R, *FCGR3A* F158V polymorphisms and clinical response of mCRC patients with adjuvant anti-EGFR mAb therapy

Population	Model	Comparison	ORR			DCR		
			No.	*P*_h_	OR(95%CI)	No.	*P*_h_	OR(95%CI)
***FCGR2A* H131R**								
**Overall**	Co-dominant	HR vs. HH	738	0.456	1.136(0.815–1.584)	297	0.987	1.244(0.732–2.112)
		RR vs. HH	463	0.234	1.308(0.852–2.009)	185	0.077	1.035(0.307–3.495)
	Dominant	HR/RR vs. HH	1857	0.389	1.156(0.919–1.453)	1333	0.307	0.993(0.780–1.265)
	Recessive	RR vs.HR/RR	933	0.283	1.269(0.908–1.773)	370	0.057	0.883(0.285–2.732)
	Over-dominant	HR vs. HH/RR	904	0.358	0.993(0.744–1.325)	370	0.79	1.242(0.805–1.916)
	Allele	R vs. H	1808	0.116	1.180(0.959–1.452)	740	0.002	0.780(0.414–1.471)
***KRAS* wild**	Co-dominant	HR vs. HH	393	0.391	1.234(0.831–1.835)	123	0.941	1.459(0.782–2.722)
		RR vs. HH	246	0.184	1.234(0.771–1.974)	74	0.178	1.623(0.646–4.076)
	Dominant	HR/RR vs. HH	1110	0.316	1.247(0.960–1.619)	790	0.671	1.115(0.819–1.518)
	Recessive	RR vs.HR/HH	500	0.14	1.193(0.752–1.892)	151	0.096	1.175(0.291–4.748)
	Over-dominant	HR vs. HH/RR	471	0.175	1.079(0.740–1.571)	151	0.422	1.323(0.622–2.815)
	Allele	R vs. H	942	0.043	1.10(0.692–1.748)	302	0.084	1.304(0.765–2.224)
***FCGR3A* V158F**								
**Overall**	Co-dominant	FV vs. FF	834	0.583	0.805(0.592−1.096)	283	0.15	0.937(0.567–1.550)
		VV vs. FF	561	0.644	0.881(0.565–1.375)	193	0.007	1.311(0.292–5.897)
	Dominant	FV/VV vs. FF	1026	0.113	0.880(0.667–1.162)	383	0.038	0.916(0.576–1.458)
	Recessive	VV vs. FV/FF	1983	0.874	0.987(0.743–1.311)	1331	0.041	0.897(0.407–1.973)
	Over-dominant	FV vs. FF/VV	977	0.735	0.855(0.643–1.138)	339	0.752	0.549–1.390
	Allele	V vs. F	1954	0.351	0.866(0.702–1.068)	678	0.013	1.019(0.541–1.919)
***KRAS* wild**	Co-dominant	FV vs. FF	404	0.61	0.851(0.572–1.266)	119	0.052	0.898(0.269–2.992)
		VV vs. FF	266	0.935	1.153(0.640–2.077)	81	0.072	1.302(0.286–5.940)
	Dominant	FV/VV vs. FF	471	0.605	0.889(0.614–1.287)	154	0.024	0.987(0.284–3.429)
	Recessive	VV vs. FV/FF	1154	0.961	1.089(0.779–1.522)	815	0.283	0.710(0.483–1.043)
	Over-dominant	FV vs. FF/VV	471	0.528	0.887(0.602–1.306)	154	0.268	0.708(0.332–1.511)
	Allele	V vs. F	942	0.707	0.987(0.744–1.311)	308	0.033	1.121(0.403–3.119)
***FCGR* combined**								
**Overall**		HH or VV vs. R/F	160	0.126	1.698(0.762–3.784)	160	0.107	1.012(0.524–1.955)
		RR or VV vs. H/F	115	0.787	0.684(0.210–2.222)	115	0.001	4.103 (0.108–156.599)

**Abbreviation:** mCRC: metastatic colorectal cancer; *P*_h_: result of heterogeneity test; ORR: objective response rate; DCR: disease control rate; OR: odds ratio; CI: confidential interval;

**Table 5:** Meta-analysis results of the association of *FCGR2A* H131R, *FCGR3A* F158V polymorphisms and clinical survival of mCRC patients with adjuvant anti-EGFR mAb therapy

Population	Model	Comparison	PFS			OS		
			No.	*P*_h_	MSR(95%CI)	No.	*P*_h_	MSR(95%CI)
***FCGR2A* H131R**								
**Overall**	Co-dominant	HR vs. HH	739	<0.001	1.111 (0.909–1.358)	573	<0.001	1.156 (0.929–1.438)
		RR vs. HH	466	<0.001	0.936 (0.704–1.244)	364	<0.001	0.864 (0.616–1.212)
	Dominant	HR/RR vs. HH	1363	<0.001	0.993 (0.782–1.262)	1176	0.822	0.946 (0.894–1.002)
***KRAS* wild**	Co-dominant	HR vs. HH	471	<0.001	1.092 (0.892–1.338)	348	0.062	1.116 (0.942–1.323)
		RR vs. HH	285	0.015	0.953 (0.783–1.159)	210	0.818	0.933 (0.815–1.068)
	Dominant	HR/RR vs. HH	980	<0.001	0.974 (0.778–1.220)	821	0.044	1.056 (0.890–1.253)
***FCGR3A* V158F**								
**Overall**	Co–dominant	FV vs. FF	789	<0.001	0.943 (0.767–1.159)	617	<0.001	1.094 (0.907–1.320)
		VV vs. FF	514	<0.001	0.896 (0.679–1.181)	405	<0.001	**0.733 (0.578–0.930)**
	Dominant	FV/VV vs. FF	311	0.027	**0.680 (0.549–0.842)**		-	-
	Recessive	VV vs. FV/FF	1077	<0.001	1.292 (0.480–3.479)		-	-
***KRAS* wild**	Co-dominant	FV vs. FF	478	<0.001	1.054 (0.872–1.275)	360	<0.001	1.128 (0.842–1.510)
		VV vs. FF	323	<0.001	1.061 (0.733–1.536)	244	<0.001	0.766 (0.521–1.126)
	Dominant	FV/VV vs. FF	283	0.12	**0.728 (0.648–0.818)**		-	-
	Recessive	VV vs. FV/FF	712	<0.001	1.382 (0.612–3.12)		-	-

**Abbreviation:** mCRC: metastatic colorectal cancer; *P*_h_: result of heterogeneity test; PFS: progression-free survival; OS: overall survival; MSR: median survival ratio; 95%CI: 95% confidential interval.

## DISCUSSION

This study, to the best of our knowledge, is the first report that combined a retrospective study and a comprehensive meta-analysis with the largest sample size for estimating the association between *FCGR2A* H131R and *FCGR3A* V158F and clinical outcome of mCRC patients with treatment of anti-EGFR mAb based therapy. With limitation of small sample size, our retrospective study showed no significant association between *FCGR2A* and *FCGR3A* polymorphisms and clinical outcome in 82 wild-*KRAS* chemorefractory mCRC individuals treated with chemotherapy plus cetuximab. However, the robust results of meta-analysis showed that genotype FF within V158F was significant associated with a longer PFS and OS than FV/VV and VV genotype in overall and *KRAS* wild population, respectively. These findings suggested that *KRAS* wild chemorefractory mCRC individual harbored genotype FF of V158F could benefit from anti-EGFR mAb based therapy in terms of PFS and OS, and the genotype could be used as a genetic biomarker to predict clinical prognosis of wild-*KRAS* mCRC patients with adjuvant treatment of anti-EGFR mAb.

ADCC is considered as an important mechanism for the antitumor effect of anti-EGFR mAb in mCRC patients [13, 32]. Cetuximab, an IgG1 monoclonal antibodies, can bind with EGFR protein of tumor cell by its Fab fragment, and its Fc fragment engages FCGR on an immune cell to activate the process of ADCC, eventually resulting in attacking and eliminating the cetuximab-coated tumor cell. Alternation of amino acid at specific binding-domain of Fc fragment of IgG can affected FCGR2A and FCGR3A binding affinity *in vitro* and *in vivo* [33–35]. H131A and V158F are located at the extracellular ligand-specific binding domain of *FCGR2A* and *FCGR3A*, respectively. *FCGR2A*-H allele and *FCGR3A*-V allele and VV genotype were associated with higher affinity to lgG2, lgG1 and lgG3 than others [35–37], respectively. Thus, the two common polymorphisms within *FCGR2A* and *FCGR3A* affected their receptor's specific binding-domain of IgG, leading to different antitumor effect in mCRC patients with anti-EGFR mAb based therapy.

Several studies concerning *FCGR* polymorphisms and efficacy of trantuumab and rituximab demonstrated that *FCGR2A* H131R and *FCGR3A* V158F could predict clinical efficacy and survival of patients with breast cancer and lymphoma, respectively [17, 38]. Cohort study concerned mCRC supported the hypothesis that *FCGR2A* H131R and *FCGR3A* V158F were associated with clinical outcome of mCRC patients with cetuximab in a small sample size [30]. Whereas, a large cohort study containing 1123 chemorefractory mCRC patients failed to support the significant results [18]. In our retrospective study, we didn't observe the significant association between *FCGR2A* H131R and *FCGR3A* V158F and clinical outcome in chemotherapy plus cetuximab treated mCRC patients in terms of ORR, DCR, PFS and OS in retrospective study. Moreover, H131R within *FCGR2A* wasn't associated with ORR, DCR, PFS and OS in overall and *KRAS* wild subgroups in the meta-analysis, indicating that the polymorphism of *FCGR2A* might be not involved in anti-EGFR mAb induced ADCC effect and it could not predict clinical outcome of cetuximab based therapy in wild-*KRAS* mCRC patients. Nonetheless, V158F within *FCGR3A* was significantly associated with PFS in dominant genetic model in overall and *KRAS* wild populations and genotype VV of the loci was significantly associated with a shorter OS in overall population in the meta-analysis. These findings suggested that V158F of *FCGR3A* was involved in clinical survival of anti-EGFR mAb treated mCRC individual, and genotype FF could be used as a prognostic biomarker to predict survival of anti-EGFR mAb treated mCRC patients in overall population, especially *KRAS* wild carrier. Our finding is in agreement with previous studies conducted by Zhang et al and Pander et al [25, 30], respectively, but against our speculation and inconsistent with the results of other studies [18, 19, 23].

The possible reason why genotype FF of V158F within *FCGR3A* was associated a longer PFS and OS compared with genotype VV and VV/VF remains partly understand. Included individuals in our retrospective study and eligible studies in meta-analysis were all chemorefractory mCRC patients, and long-time's regular chemoradiotherapy and increasing burden of CRC leaded to a gradual decreased immunologic surveillance [18, 26]. NK cell was generally scarce within CRC tumor tissues, on the contrary, normal level of NK cell was examined in adjacent normal mucosa [39]. Meanwhile, a significant reduction in the percentage of NKG2D+NK cell was observed in peripheral blood of metastatic colon cancer patients [40]. Additionally, tumor-associated macrophage (TAM) is one of the most frequently found immune cells in the tumor microenvironment [41]. Although genotype VV of V158F displayed a significant higher anti-EGFR mAb-triggered ADCC in peripheral blood mononuclear cells *in vitro* [23]. However, a possible mechanism is that the high-affinity allele V of V158F within *FCGR3A* might contribute to decreased ADCC-triggered by NK cell [18, 26], but through cross-linking of the FCGR to increase activation of TAM in tumor microenvironment by anti-EGFR mAb. Activated TAM releases large amounts of pro-angiogentic and pro-metastatic regulators to facilitate angiopoiesis, matrix breakdown, tumor cell motility and promote tumor growth, leading to poor prognosis [41, 42].

In conclusion, we fail to find the significant association between *FCGR2A* H131R and clinical outcome in *KRAS* wild mCRC individuals with adjuvant cetuximab therapy, but *KRAS* wild chemorefractory mCRC individual harbored genotype FF of V158F can benefit from cetuximab based therapy in terms of PFS and OS, and it may be a prognostic factor to evaluate the clinical survival of wild-*KRAS* mCRC patients undergoing anti-EGFR mAb therapy. With limitation of the study, multi-central, well designed prospective studies with large sample size are warrant to further validate our findings.

## MATERIALS AND METHODS

### Patients

Patients with mCRC who diagnosed and treated in Nanjing First Hospital and Pingxiang People's Hospital between 2007 May and 2014 December were enrolled in our retrospective study. All of them were histologically proven TNM-IV stage and harbored with wild-*KRAS*. The included individuals were treated with cetuximab plus conventional chemotherapy regimen such as CapeOX, FOLFIR1 and FOLFOX. Cetuximab was used 400mg/m^2^ dose at first, then 250mg/m^2^ subsequently, 21 day a cycle, and treated until cancer progression or unacceptable toxicity. This study was approved by the Institution Ethics Commission of Southeast University, and all included participants were signed informed consents.

### Efficacy evaluation and following-up

In accordance with the Response Evaluation Criteria In Solid Tumors Criteria 1.0 (RECST 1.0), the cancer response to cetuximab therapy was evaluated every month at the time of hospitalization and the evaluated results were defined as complete response (CR), partial response (PR), stable disease (SD) and progressive disease (PD), respectively. Progression-free survival (PFS), overall survival (OS) and objective response rate (ORR) and disease control rate (DCR) were used as endpoints in our study. PFS and OS were calculated from the first date of cetuximab usage to the time of disease progression and death, respectively. Relevant data (sex, age, treatment, *KRAS* status, and response evaluated results) were collected from the cases' medical records.

### PCR amplification and genotyping

Human genomic DNA was extracted from paraffin-embedded CRC tissues or EDTA anti-coagulated peripheral blood samples using TIANamp Genomic DNA Kit (TIANGEN, Beijing, China) and stored at −80°C till detection. Taqman-genotyping real-time PCR were selected to detect genotypes of *FCGR2A* H131R and *FCGR3A* V158F using ABI7500 fluorescence quantitative PCR system (Applied Biosystems, Foster City, USA). The detail probe, primer sequence, PCR protocol and detection were in accordance to the study reported by Norton et al [38].

### Meta-analysis

In order to enhance statistical power of the study, a meta-analysis including all eligible studies was conducted to further confirm our results. Relevant article was screened in the Pubmed, Web of SCI and Wanfang databases in English and Chinese using search terms of “*FCGR2A/3A* and cetuximab or CRC”, “rs1801274/rs396991 and cetuximab or CRC”, and “ H131R/V158F and cetuximab or CRC” dating end up to March of 2015. In addition, a manual searching in reference of relevant articles was carried out to obtain substantial studies. Relevant articles were identified as eligible study in accordance with following including criteria: (1) retrospective or prospective study was concerned *FCGR2A/3A* polymorphisms and clinical outcome of chemorefractory mCRC cases treated with adjuvant cetuximab”; (2) efficacy evaluation criteria was in accordance with RECST 1.0/1.1; (3) studies provided median survival time, number of cases, genotype distributions, ORR, DCR. On the contrary, review, correspondence, letter, meta-analysis, case report or studies without providing relevant data were excluded from the study. Relevant study search, eligible study identification, data extraction and statistics were conducted by two independent investigator (Hou-Qun Ying and Feng Wang) and any discrepancies were discussed to reach consensus or made final decision by the third investigator (Xiao-Lin Chen).

### Statistical analysis

The genotype frequencies of *FCGR2A* H131R and *FCGR3A* V158F in all cases were calculated by counting. Pearson χ^2^ test was selected to evaluate association between the loci genotypes and response to cetuximab based treatment, and *P* < 0.05 were considered statistically significance. Kaplan-Meier curve with log-rank test and backward elimination multivariate Cox regression analysis were used to determine the influence of the *FCGR2A* and *FCGR3A* polymorphisms on PFS and OS, and the significant *P*-value was set at 0.05. Q test and estimated I^2^ were used to assess the heterogeneity between eligible studies, and *P*_h_ < 0.1 or I^2^ > 50% was considered as exist of significant heterogeneity. Overall effect of the meta-analysis was evaluated using Z test in the fixed model (*P*_h_ > 0.1) or the random model (*P*_h_ < 0.1) according to the heterogeneity test and *P*_z_ < 0.05 showed a statistical significance. Begg's funnel plot was selected to estimate the possible publication bias, and asymmetric funnel plot was considered as the existence of publication bias. All statistical analyses were performed using the SPSS statistical 17.0 (SPSS Inc., Chicago, IL) and Stata 11.0 software (Stata Corporation, College Station, TX).

## SUPPLEMENTARY TABLE


