# Supplementary material for: FCGR2A, FCGR3A polymorphisms and therapeutic efficacy of anti-EGFR monoclonal antibody in metastatic colorectal cancer
Source: Oncotarget. 2015 Aug 19;6(29):28071–83. doi: 10.18632/oncotarget.4872 (PMC4695045; doi:10.18632/oncotarget.4872)
Supplement: Supplementary file 1 [file oncotarget-06-28071-s001.pdf]

SUPPLEMENTARY TABLE

Supplementary Table S1: The baseline characteristics of the study included in the meta-analysis

Author	Country	Race	Design	Patient	*KRAS* status	Antibody	Locus	Detection	Criteria	Clinical outcome
Zhang 2007	USA	Caucasian	Prospective	39 chemorefractory mCRC cases	Wild/ mutated	Cetuximab	H131R/ V158F	AS-PCR	RECIST 1.0	CR, PR, SD, PD, PFS, OS
Bibeau 2009	France	Caucasian	Retrospective	69 chemorefractory mCRC cases	Wild/ mutaed	Cetuximab	H131R/ V158F	Sequencing, AS-PCR	RECIST 1.0	CR, PR, SD, PD, PFS
Paez 2010	Spain	Caucasian	Retrospective	104 chemorefractory mCRC cases	Wild/ mutated	Cetuximab, panitumumab	H131R/ V158F	TaqMan	RECIST 1.0	CR, PR, PD, SD, PFS
Zhang 2010	USA	Caucasian	Retrospective	83 chemorefractory mCRC cases	Wild/ mutated	Cetuximab,	H131R, / V158F	PCR-RFLP	not concern	Response, no-response, PFS, OS
Pander 2010	Holland	Caucasian	Prospective	246 chemorefractory mCRC cases	Wild	Cetuximab	H131R/ V158F	TaqMan, sequencing	RECIST 1.0	PFS
Dahan 2011	France	Caucasian	Retrospective	58 chemorefractory mCRC cases	Wild	Cetuximab	H131R/ V158F	PCR-RFLP	RECIST 1.1	CR, PR, PD, SD, PFS, OS
Etienne 2011	France	Caucasian	Prospective	52 chemorefractory mCRC cases	Wild/ mutated	Cetuximab	H131R/ V158F	PCR-RFLP	RECIST 1.1	CR, PR, PD, SD, PFS, OS
Calemma 2012	Itary	Caucasian	Retrospective	74 chemorefractory mCRC cases	Wild	Cetuximab, panitumumab	H131R/ V158F	Sequencing	RECIST 1.0	CR, PR, PD, SD, PFS
Park 2012	Korea	Asian	Retrospective	107 chemorefractory mCRC cases	Wild/ mutated	Cetuximab	H131R/ V158F	TaqMan	RECIST 1.1	Response, no-response, PFS, OS
Saito 2013	Japan	Asian	Retrospective	32 chemorefractory recurrentor mCRC cases.	Wild	Cetuximab,. panitumunab	H131R/ V158F	TaqMan	RECIST 1.1	CR, PR, PD, SD, PFS, OS
Geva 2014	European, Japan, USA	Asian, Caucasian	Retrospective	1123 chemorefracotry mCRC cases	Wild/ mutated	Cetuximab	H131R/ V158F	Taqman	RECIST 1.0	CR, PR, PD, SD, PFS, OS
Inoue 2014	Japan	Asian	Prospective	57 chemorefractory mCRC cases	Wild	Cetuximab	H131R/ V158F	Sequencing	RECIST 1.0	CR, PR, PD, SD, PFS, OS
Kjersem 2014	Norway	Caucasian	Prospective	571 chemorefractory mCRC cases	Wild/ mutated	Cetuximab	H131R/ V158F	Taqman	RECIST 1.0	Response, no-response, PFS, OS
Negri 2014	Itary	Caucasian	Retrospective	86 chemorefractory mCRC cases	Wild/ mutated	Cetuximab	H131R/ V158F	Sequencing	RECIST 1.1	CR, PR, PD, SD, TTP, OS
Ying 2015	China	Asian	Retrospective	82 chemorefractory mCRC cases	Wild	Cetuximab	H131R/ V158F	Taqman	RECIST 1.1	CR, PR, PD, SD, PFS, OS

Abbreviation: mCRC: metastatic colorectal cancer; AS-PCR: allele specific-polymerase chain reaction; PCR-RFLP: polymerase chain reaction-restrained fragment length polymorphism; RECIST: response evaluation criteria in solid tumors; CR: complete response; PR: partial response; PD: progressive disease; SD: stable disease; TTP: time to progression; PFS: progression-free survival; OS: overall survival.
